# Response heterogeneity: Challenges for personalised medicine and big data approaches in psychiatry and chronic pain

**DOI:** 10.12688/f1000research.13723.2

**Published:** 2018-03-01

**Authors:** Agnes Norbury, Ben Seymour

**Affiliations:** 1 Computational and Biological Learning Laboratory, Department of Engineering, University of Cambridge, Cambridge, CB2 1PZ, UK; 2 Center for Information and Neural Networks, National Institute of Information and Communications Technology, Osaka, 565-0871, Japan

**Keywords:** personalised medicine, big data, machine learning, psychiatry, chronic pain, individual differences, response heterogeneity, clinical trial design

## Abstract

Response rates to available treatments for psychological and chronic pain disorders are poor, and there is a substantial burden of suffering and disability for patients, who often cycle through several rounds of ineffective treatment. As individuals presenting to the clinic with symptoms of these disorders are likely to be heterogeneous, there is considerable interest in the possibility that different constellations of signs could be used to identify subgroups of patients that might preferentially benefit from particular kinds of treatment. To this end, there has been a recent focus on the application of machine learning methods to attempt to identify sets of predictor variables (demographic, genetic, etc.) that could be used to target individuals towards treatments that are more likely to work for them in the first instance.

Importantly, the training of such models generally relies on datasets where groups of individual predictor variables are labelled with a binary outcome category − usually ‘responder’ or ‘non-responder’ (to a particular treatment). However, as previously highlighted in other areas of medicine, there is a basic statistical problem in classifying *individuals *as ‘responding’ to a particular treatment on the basis of data from conventional randomized controlled trials. Specifically, insufficient information on the partition of variance components in individual symptom changes mean that it is inappropriate to consider data from the active treatment arm alone in this way. This may be particularly problematic in the case of psychiatric and chronic pain symptom data, where both within-subject variability and measurement error are likely to be high.

Here, we outline some possible solutions to this problem in terms of dataset design and machine learning methodology, and conclude that it is important to carefully consider the kind of inferences that particular training data are able to afford, especially in arenas where the potential clinical benefit is so large.

## Introduction

The proportion of patients who respond to available treatments for psychological and chronic pain disorders is often low. For example, in major depression, roughly 40% of individuals experience a ‘clinically significant’ response (decrease in symptom severity score above some minimum value) over the course of treatment (e.g. 1, 2). Similarly, a recent meta-analysis of available pharmacotherapies for neuropathic pain found estimates of ‘number needed to treat’ (number of patients needed to be treated to prevent one additional adverse clinical outcome) for *effective* treatments ranged from 4–10, indicating poor response rates ^3^. For patients, this often means a lengthy process of cycling through different treatment options, in a sequence that may be significantly influenced by non-clinical concerns (e.g. relative drug cost, therapist availability, local health authority guidelines), and where there may be inadequate data on the safety and effectiveness of switching regimes (e.g. 4). For psychological conditions, this process can be particularly lengthy, given the significant period of time before common pharmacological treatments are expected to take effect (e.g. 4–6 weeks to conclude a particular drug treatment is ineffective, ^4^). Together, this results in a substantial burden of suffering and disability for individuals with a diagnosis of these disorders, before (if) an effective treatment option can be found.

It is generally assumed that differential response to a particular treatment across individuals can be at least partially explained by patient heterogeneity within a certain diagnostic category – i.e. that individuals who present to the clinic with similar sets of symptoms may have different underlying pathologies. This seems a particularly reasonable assumption in the case of both mental health disorders and chronic pain, as diagnosis is often made purely on the basis of self-reported symptom checklists, and our lack of knowledge into the aetiology of these conditions means we have little opportunity for differential diagnosis. Indeed, in the case of psychiatric disorders, such as depression, diagnosis can often be made on the basis of directly contradictory symptom reports (e.g. sleeping too much *vs* sleeping too little), and there may be many different ways to meet diagnostic criteria (e.g. 227 possible symptom combinations for major depressive disorder, according to DSM-IV ^5^). Similarly, even patients with a diagnosis of a particular pain condition are likely to have distinct patterns of nervous system damage, involving multiple pathways (e.g. 6), and definitions of chronic pain itself can vary dramatically across research groups and clinical centres ^7^.

Even if we lack insight into pathological mechanisms, it seems likely that if we are able to use some kind of predictive method to direct individuals towards treatments that are likely to be more effective for them – then even a small increase in the resulting response rate could potentially have a large effect on disease burden for individual patients. There has therefore recently been great interest in doing just this for psychiatric data, via application of supervised learning methods to large datasets of individual clinical predictors and treatment response data (see 8 for an excellent recent review of potential clinical advantages and best methodological practice in this area).

The current gold standard approach is firstly to define a set of features and targets for various machine learning algorithms to train on. In this context, features are individual difference variables that may potentially relate to future treatment outcome (clinical, demographic, physiological, genetic, behavioural, etc. information). The target variable (that the algorithm must learn to predict) is usually a binary category label, such as ‘responder’ or ‘non-responder’ (whether or not an individual has exhibited symptom improvement above some threshold level, following a particular course of treatment). Various supervised learning algorithms can then be trained on this labelled dataset (ideally using a rigorous cross-validated approach), and assessed in terms of their predictive accuracy on independent ‘unseen’ (during model training) data. Finally, the best model can be brought forward to a randomised controlled trial framework, where treatment allocation by current clinical guidelines could be compared to algorithm-assisted treatment assignment ^8^.

This approach is highly attractive, as the potential clinical gains from even a small increase in likelihood of treatment response for a particular individual are large. However, across the field of medicine in general, attempts to make pursue a personalised medicine approach have not fulfilled their initial promise – with relatively few reaching the clinic (e.g. 9). Here, we explore a basic statistical issue that may limit the effectiveness of this process – i.e. the validity of distinguishing between treatment ‘responders’ and ‘non-responders’ in the first place. We further discuss the reasons why this problem may be particularly acute in the case of available data regarding psychiatric disorders and chronic pain conditions, and some potential solutions.

## The problem of response heterogeneity

The problem of properly identifying response heterogeneity, i.e., reliably distinguishing between responders and non-responders to a particular treatment, on the basis of randomised controlled trial (RCT) data, has previously been highlighted across various fields of medicine ^10– 12^. If not properly addressed, this constitutes an absolute limit on the effectiveness of predictive models at the level of input or training data, thereby limiting their future clinical usefulness.

The issue is best illustrated by considering the nature of data collected during RCTs, and the kind of inference this process affords. The foundation of an RCT is that the mean effect of an intervention (e.g. active drug treatment) is derived by comparing what happened, on average, to the (randomly allocated) participants in the intervention group to what happened, on average, to participants in the control (e.g. placebo) arm. The random allocation of participants to the intervention *vs* control arms allows the control group to function as an illustration of what we might have expected to occur in the intervention group, had they *not* received the active treatment – in turn allowing us to draw conclusions about the overall (average) effects of the treatment itself ^12^. Crucially, we can draw this inference only by direct comparison to the control arm data.

This basis of an RCT means that we cannot identify responders and non-responders by considering individuals in the intervention group *alone*. In other words, we it is hard to legitimately label an individual who received a particular active treatment as a ‘responder’ (or not), because we do not know what would have happened to that particular individual if they had been in the comparator arm ^10^. This kind of information is very hard to obtain at the individual ( *cf* the group) level, as there is no good way to obtain a control observation. Formally, to properly infer whether a particular participant responded or didn’t respond to a particular treatment, we would require knowledge of what would have happened if a key event (treatment administration) both *did* and *did not* occur (a form of counterfactual reasoning), which is not possible in the real world ^11^.

Although the underlying RCT datasets almost always consist of at least two arms (e.g. active treatment *vs* placebo), machine learning algorithms employed to predict psychiatric treatment response are usually trained on active treatment arm data alone – without reference to control arm symptom changes (e.g. 13, 14). Unless sufficient care is taken, these kinds of predictive models may therefore be the inferential equivalent of single arm trials, and the resultant categorisation of symptom change scores may be unduly influenced by sources of variance causally unrelated to true treatment response.

### A particularly acute issue for psychiatric and chronic pain datasets?

Variability of change (e.g. *t*_2_ – *t*_1_ symptom score) in the intervention arm is not a true estimate of variability in treatment response, because it includes components of within-subject variation and measurement error ^10^. Even if measurement error is small (i.e. we can precisely measure the outcome variable of interest), for many medical interventions, the outcome variable will depend on a complex interplay of biological factors (e.g. time of day, stress level, etc.), and so within-subject variability will be relatively high. This means that the reliability of within-subject measurements across time points can be somewhat poor, and large variation in changes between study time points may be evident − even where there is no true individual difference in treatment response.

Unfortunately, for psychiatric and chronic pain symptom data, both measurement error and within-subject variation are likely to be high. Although self-reported symptom levels are considered the gold standard outcome measure for both psychiatric disorders and chronic pain conditions ^15^, reliability is limited by factors such as cognitive capacity and level of insight for patient-rated measures (e.g. 16), and by interviewer skill and inter-rater agreement for clinician-rated measures (e.g. 17– 19). Further, these classes of disorders represent episodic, chronically relapsing conditions, which will likely contribute to large within-subject variation, particularly at typical RCT follow-up timescales (often around 6 months–1 year; *cf* e.g. median duration of a depressive episode of ~20 weeks ^20^). The greater the variation in outcome due to these sources, the harder to it will be to detect true individual differences in treatment response, under a conventional RCT design.

A further problem in predicting true response heterogeneity is susceptibility of symptom change data to regression to the mean and mathematical coupling artefacts ^21, 22^. Regression to the mean refers to the phenomenon whereby if an individual is selected on the basis of having an extreme measurement value at time point one, their second measurement value will, on average, be closer to the mean of the population distribution (due to the influences of measurement error and normal within-subject variation). A corollary of this effect is that *t*_1_ severity is often a significant covariate of change in symptom score between *t*_1_ and *t*_2_, – meaning that individuals with higher initial scores may appear to show the greatest improvement in symptom levels at follow-up, even when the true magnitude of change does not vary across individuals (see 10 for a worked example). The fact the *t*_1_ score is used to calculate both baseline and change scores (i.e., that they are mathematically coupled) results in further inflation of this relationship (see 22). Care should therefore be taken when key predictors in response algorithms closely index *t*_1_ severity, as this may result in a poorly generalising model. However, in previous studies based on psychiatric datasets, baseline severity score is usually included among the features used to train response prediction algorithms (e.g. 13, 14, 23).

These factors may help explain why previous attempts to apply machine learning approaches to outcome prediction in psychological disorders have thus far had limited success in terms of out-of-sample (unseen data) classification. For example, a recent methodologically rigorous trial aiming to predict significant response (remission) following treatment with a particular antidepressant drug achieved only ~60% classification accuracy when the model was applied in external validation datasets ^14^. However, as previously noted, tools with only modest true predictive value may still have reasonably high clinical utility compared to current best practice ^8^; therefore this is still an approach very much worth pursuing.

## Potential solutions

### Clinical trial design

The problem of identifying true response heterogeneity is a problem of appropriately partitioning variance components in observed outcomes ^11^. The ability to identify differential response to particular treatments in different individuals can be achieved by replication of observations at the level at which the differential response is claimed (i.e., that particular treatment in that particular individual). Differential treatment response (i.e., identification of patient-by-treatment interactions) can therefore be identified by use of repeated period cross-over designs – a form of trial where each participant receives both placebo and active treatments more than once ^11^. However, in practice, these designs are rare, as they are likely to be impractical (prohibitively lengthy and expensive) and/or unethical. This kind of design also assumes that treatments wash out fully between administrations, which might not be reasonable for some interventions (e.g. psychological therapies) ^24^.

### Training data definition and selection

An alternative approach is to improve the way data from existing RCTs is used to train predictive models. For example, it has been suggested that the uncertainty in each individual’s ‘response’ (change in symptom score in the active treatment group) could be expressed as a confidence interval by reference to the standard deviation of the change scores in the control (placebo) group multiplied by the appropriate value from the *t* distribution (e.g. individual change score ± 1.96*SD of control arm changes for a 95% CI, see 24). The probability that any given individual in the intervention group is a true responder (true change score is greater than the minimum clinically significant change) can then be derived from individual CIs using a Bayesian approach ^10^. Appropriate supervised learning algorithms could then be trained to predict (continuous) treatment response probability, as opposed to dividing individuals into binary response categories (e.g. using Gaussian process regression ^25^). This approach could also be used to predict individual probability of *harm* (worsening of outcome measure above some minimum clinically important threshold) in response to a particular treatment. Some researchers have suggested that comparing the variances of symptom change data between active treatment and control arms, in order to detect whether there is clinically significant heterogeneity in response to a particular treatment in the first place, should be a pre-requisite for these kind of analyses ^10^.

It also may be important to think carefully about the nature of the predictors (features) included in supervised learning model training data – as those that reference initial clinical severity may be vulnerable to regression to the mean-related artefacts. Under a traditional statistical framework, an effective way of dealing with these artefacts is to include baseline scores as covariates in models of symptom change data (i.e., conduct an ANCOVA). This approach can then be used to test if a given between-subjects variable is a significant modifier of treatment effect by adding it to the model (providing measurement error is sufficiently low ^24^). An interesting issue is that in machine learning, there is not really an equivalent concept to ‘covariates of no interest’ – rather, model features are usually selected purely on the basis of their predictive capacity. One recent paper that explicitly addresses this problem comes from Rao and colleagues, who propose a method for removing known confounds from predictive models based on functional imaging data. Rao *et al.* suggest that one solution is to first fit linear models to each image feature using the confound variables as predictors, then consider the residuals of this model to be ‘adjusted’ data − suitable to be used as input features for a confound-controlled predictive model ^26^. There are also statistical methods that have proposed to correct for regression to the mean when simply correlating *t*_2_- *t*_1_ symptom changes with initial severity level that could be applied to training data (see 22). However, these may require additional measurements (e.g. multiple estimates of *t*_1_ value, in order to estimate measurement reliability).

### Counterfactual probabilistic modelling and other causal inference methods

When a particular experiment is not feasible, an alternative is to train models on observational (non-experimental) data that are able to make counterfactual predictions – i.e. of the outcomes that would have been observed, had we run that particular experiment. For example, Saria and colleagues have recently developed a counterfactual Gaussian process (CGP) approach to modelling clinical outcome data ^27^. The CGP is trained on observational symptom trajectory data to form a model of clinical outcomes under a series of treatments in continuous time. Crucially, the CGP is trained using a joint maximum likelihood objective, which parses dependencies between observed actions (e.g. treatments) and outcomes in order to infer the existence of *causal* relationships between the two. This feature allows the prediction of how future trajectories (symptom levels) may change in response to different treatment interventions, and has previously been shown to successfully predict real clinical data (renal health markers following different kinds of dialysis, ^27, 28^).

Thus far, the CGP has only been empirically tested as a clinical decision support tool on the same subjects from whom model training data was derived ^27^. However, it can be argued that prediction of the response of *future* patients to particular treatment options is an inference problem that does not necessarily involve counterfactual reasoning. Under these circumstances, we require a model that can infer causes that are likely to be active for *out-of-sample* data (individuals with certain features, who may not have received any treatment yet), as opposed to *in-sample* data (individuals whose clinical data a particular model was trained on, who might have showed a different response to different treatment strategies) ^29^.

This perspective raises the issue of how representative the individuals who make up a particular training dataset are of the general population (from whom future patients will be drawn). Importantly, concerns have previously been raised as to the effects of various sources of selection bias on the representativeness of participants in RCTs compared to the population at large ^30^. Specific forms of selection bias that have been identified in RCTs for psychological disorders include exclusion of individuals with comorbidities (which for many some conditions may be more common than ‘pure’ presentation), selection of less severe cases (e.g. in psychotic disorders, where ability to consent and treatment compliance may be of heightened concern), or, conversely, application of minimum severity thresholds (e.g. in mood disorders, to reduce the likelihood of spontaneous remission over the trial period) ^31, 32^. Further, methods of recruitment to RCTs (particularly the requirement to self-select into trials) may influence the distribution of various psychological traits in trial participants, prior to any further eligibility criteria being applied (e.g. 33). Although there are methods designed to mitigate the effects of sample selection bias when transferring predictive models to a different test set (see 34), it remains an open question as to whether these are sufficiently robust for successful out-of-sample treatment prediction at the individual level.

The success of causal inference modelling approaches to response prediction may therefore depend upon availability of different kinds of data to that derived from traditional RCTs – involving semi-continuous measurement of the relevant clinical outcome (both pre- and post- intervention), and gathered from more representative sources than some previous RCT datasets. Given sufficient attention to patient confidentiality and other ethical concerns, it may be possible to obtain appropriate training data from health service clinical records; however, frequency and consistency of symptom reporting may pose analytical problems (e.g. 28). The use of personal devices such as smartphones or other wearable technology to regularly self-record symptom levels may be a potential source of this kind of data in the future, given sufficient insight and patient compliance (e.g. 35).

A further important feature of predictive models derived from observational data is that they depend on explicit assumptions about the existence and causal consequences of any confounding variables present in the dataset. For example, the CGP approach requires both that there will be a consistency of outcomes between training observations and future outcomes, given a particular treatment, and that there are no important confounding variables missing from the dataset ^27^. It will therefore be necessary to carefully consider how well such assumptions are met when considering applying these kinds of models to psychological and chronic pain symptom data.

## Conclusions

The issues discussed above underline the importance of focusing on where data comes from when considering strategies for personalised medicine. In particular, it is problematic to designate individual data points from a conventional RCT design as ‘responders’ or ‘non-responders’ to a particular treatment, as symptom change scores are not adjusted for other important sources of variation. This might be particularly important when considering patients with episodic, chronically-relapsing disorders, as within-subject variability is likely to be high (and symptom measurement itself may be imprecise). One solution to this problem is to use data derived from repeated cross-over design clinical trials, although in practice these can be prohibitively difficult and/or ethically problematic. It may be possible to alleviate these issues with careful training data selection and predictive model design, but changes in the way symptom data is collected and monitored may still be required in the future in order to maximise the clinical utility of model-aided treatment selection approaches.
