# Supplementary material for: Response heterogeneity: Challenges for personalised medicine and big data approaches in psychiatry and chronic pain
Source: F1000Res. 2018 Mar 1;7:55. Originally published 2018 Jan 15. [Version 2] doi: 10.12688/f1000research.13723.2 (PMC5820606; doi:10.12688/f1000research.13723.2)
Supplement: Peer review history

# Peer review: Referee response for version 2

**DOI:** 10.5256/f1000research.15388.r31390

**Published:** 2018-03-16

**Authors:** William G. Hopkins

**Affiliations:** 1 Institute for Health and Sport, Victoria University, Melbourne, VIC, Australia

There has been no attempt to use my suggestions for making the abstract clearer, and you ignored many of my suggestions for improvement in grammar and sense and my requests for clarification in the rest of the manuscript. Never mind.

 I don't agree with your argument about repeatability.  In your response to my previous critique, you wrote: "Regarding the issue of ‘repeatability’, we would argue that since this perspective deals with the ability to predict the responses of *future *patients (i.e., individuals with similar characteristics to past ‘responders’), then repeatability of these responses is not only relevant, but vital. Although we would not deny the real benefit to an individual patient of any significant improvement in clinical outcome, if this benefit is not ‘repeatable’ in that it is reliably casually related to treatment administration (e.g. score improvement is largely due to fluctuations in symptoms that would have occurred otherwise), then it should not be taken as evidence for use of that treatment in future (similar) individuals." In other words, you think a treatment has to reproduce a benefit on a second administration in the same subject if it is to produce benefit on the first administration in a similar subject. But if the treatment produced a benefit on the first administration in one kind of subject, then there is no reason to assume it would not produce benefit on the first administration in a similar subject. Besides, we are often talking about treatments that are supposed to produce a permanent cure, in which case the question of repeatability is not an issue. But if the effect of the treatment wears off, a repeat of the same treatment could fail acutely for the same reason it failed chronically the first time (a change in the subject resulting in desensitization), so you can't extrapolate from the effect of the second treatment in the first subject to the expected effect of the treatment the first time it is administered to a similar subject.

 In the paragraph on regression to the mean, you state "The fact the *t*_1_ score is used to calculate both baseline and change scores (i.e., that they are mathematically coupled) results in further inflation of this relationship (see 22)." No, it doesn't "further inflate" regression to the mean. You get exactly the same artefact whether you use t1 to predict t2 or t2-t1. The benefit of predicting the change score is that the artefact is more obvious in scatterplots.

 Some of the new material is beyond my expertise. It reads OK.

 Find "which for many some conditions" and remove either "many" or "some". In the same paragraph, "31,32" needs to be superscripted. Whether 33 and 34 need superscripting is a problem with that method of referencing.

 "availability of different kinds of data to that derived " should be "availability of kinds of data different from those derived"

  "may be a potential" is another double doubtful you will probably not bother to fix.

I have read this submission. I believe that I have an appropriate level of expertise to confirm that it is of an acceptable scientific standard.

# Peer review: Referee response for version 1

**DOI:** 10.5256/f1000research.14907.r30529

**Published:** 2018-02-20

**Authors:** Ricardo Silva

**Affiliations:** 1 The Alan Turing Institute, London, UK

I would like to thank the authors for the informative review of the many difficulties pertinent to modeling the treatment of psychiatric disorders. My main take will be on the statistical and machine learning aspects, causal inference in particular, as I do not have a clinical background.

 With the availability of larger sources of data, it is reasonable to ask what can be leveraged in order to better predict how individual patients will respond to particular treatments. This raises questions of causal inference, as treatments are meant to be interventions that will (or are expected to) change the condition of a patient. The article properly addresses factors that make this challenging, including measurement error. I do not have much to comment on measurement error as this goes into the specifics of the domain, but I would like to second the authors' warning on how important this issue is. Even different ways of executing the data collection protocol in different environments (e.g., the way questionnaires are applied) can have an influence on what response distribution we obtain in the end.

 I will focus instead on what it means to perform counterfactual reasoning, and which challenges in performing it are warranted. I share much of the view of Dawid ^1^, that many problems of causal inference are decision-theoretical rather than genuinely counterfactual. In fact, counterfactuals are incompatible with out-of-sample problems, if only because it is impossible to be "contrary to a fact" that has not happened yet and can still happen. In the classical statistical approach for causal inference, the motivation is intrinsically an in-sample problem, where the randomness is all in the treatment assignment: we are dealing with what would have happened to a particular group of people, at a particular time, at a particular environment, had treatment assignments been different ^2^. In the questions raised by the authors, we are interested in what *will* happen to a patient given a treatment coming from a set of two or more choices (to follow one psychiatric treatment, or an alternative, or none etc.), not what *would have happened* had things been different. This is a predictive policy question, not unlike what is found in contextual bandits (but notice that, unlike contextual bandits, we are not necessarily interested in exploration-exploitation trade-offs, but on the assessment of a policy that maps features of an individual to an action). This boils down to evaluating hypothetical actions, and picking the most beneficial one according to some risk/utility function. This is still a causal inference question as it concerns the effects of actions, but it does not need to be framed as counterfactual reasoning as the notion of rewinding the clock to apply a different treatment to an individual is never on the cards when assessing out-of-sample treatment recommendation. Put simply, the estimand does not involve counterfactuals.

 How to obtain such a model is however a nontrivial question and requires much work, which I briefly discuss below in the context of the article. As a final remark before continuing, I will say that, unlike Dawid, I have no issues on using counterfactual models to address hypothetical questions. By the end of the day, there are many equivalent languages to express causal assumptions, and we should be free to choose our "syntactic sugar" as it is seen fit, as long as we know what the limitations of our models are. The following is agnostic to whether counterfactuals have motivated the model or some other predictive counterfactual-free causal approach has been used instead.

 So, what are the challenges of causal inference for heterogeneous effects? It is still the case that RCTs are much limited in this scenario: it is one thing to use a RCT so show that there exists a group of people which at particular time and at a particular environment would have shown different responses had treatment assignment been different. It is a different ballgame to extract meaningful predictive power of a dataset if the sample is not representative of the target population. This sample selection bias, where volunteers of a RCT are very likely not to be representative, is pervasive in many sciences. To the best of my understanding, this is also the case in psychiatric research. Some mitigation can be done to transfer some conclusions to the test set of interest ^3^ by tapping into some aspects of the process that remain invariant out-of-sample. Unfortunately, this may not be enough, and attending to the needs of many individuals of interest may require unwarranted extrapolations from the training set. Although RCTs are extremely desirable for causal inference, as a well-designed trial will remove unmeasured confounding, the selection bias that is natural in studies with human subjects may be too strong for its conclusions to be applicable to a large fraction of out-of-sample personalised treatments. I'm particularly skeptical of putting any effort on cross-over designs: those have the shortcomings of RCTs (sample not being representative), while adding assumptions such as "treatments wash out fully between administrations" which seem unbelievable to me in the context of psychiatric research, while the motivation (estimating counterfactuals) is unnecessary for the ultimate goal of deciding among the initial treatment options for out-of-sample patients.

 A promising venue is to exploit observational data, under the provision that we understand its many limitations. We need causal assumptions about which sources of confounding exist and how to remove them, and to which extent a RCT may provide information on the degree of confounding for patient profiles observed in the general population (via the observational data), but which are far from those found in the RCT sample.  While the machine learning literature has done much in terms of providing ways of combining data from a set of experiments and observations ^4^^,^^5^, they put much emphasis on finding causal networks as opposed to reliably estimating heterogeneous effects. This remains open, and with the added difficulty of dealing with assumptions about measurement error. Hopefully we will see this raising research questions for those up to the challenge, and with the motivation of improving the lives of the many who rely on our better understanding of psychiatric treatments and their effectiveness.

I have read this submission. I believe that I have an appropriate level of expertise to confirm that it is of an acceptable scientific standard.

## References

[1] DawidA: Causal Inference Without Counterfactuals. *Journal of the American Statistical Association*.2000;95(450) : 10.2307/2669377 10.2307/2669377

[2] RosenbaumP: Observation and Experiment. *Harvard University Press*.2017;

[3] BareinboimEPearlJ: Causal inference and the data-fusion problem. *Proceedings of the National Academy of Sciences*.2016;113(27) : 10.1073/pnas.1510507113 7345-7352 10.1073/pnas.1510507113 PMC494150427382148

[4] CooperGFYooC: Causal discovery from a mixture of experimental and observational data. *Morgan Kaufmann Publishers Inc.(San Francisco, CA)*.1999;116-125

[5] SachsKPerezOPe'erDLauffenburgerDANolanGP: Causal protein-signaling networks derived from multiparameter single-cell data. *Science*.2005;308(5721) : 10.1126/science.1105809 523-9 10.1126/science.1105809 15845847

# Response

**Published:** 2018-02-27

**Authors:** Agnes Norbury

**Affiliations:** Department of Engineering, University of Cambridge, UK

Thank you for taking the time to review this manuscript, and for providing a thoughtful meditation on individual response prediction from a casual inference perspective. We have updated the manuscript to touch upon some of the issues you have raised (please see below):

“Counterfactual probabilistic modelling **and other causal inference methods**

When a particular experiment is not feasible, an alternative is to train models on observational (non-experimental) data that are able to make counterfactual predictions – i.e. of the outcomes that would have been observed, had we run that particular experiment. For example, Saria and colleagues have recently developed a counterfactual Gaussian process (CGP) approach to modelling clinical outcome data ^27 ^. The CGP is trained on observational symptom trajectory data to form a model of clinical outcomes under a series of treatments in continuous time. Crucially, the CGP is trained using a joint maximum likelihood objective, which parses dependencies between observed actions (e.g. treatments) and outcomes **in order to infer the existence of *causal* relationships between the two**. This feature allows the prediction of how future trajectories (symptom levels) may change in response to different treatment interventions, and has previously been shown to successfully predict real clinical data (renal health markers following different kinds of dialysis, ^27, 28 ^).

**Thus far, the CGP has only been empirically tested as a clinical decision support tool on the same subjects from whom model training data was derived [27]. However, it can be argued that prediction of the response of *future *patients to particular treatment options is an inference problem that does not necessarily involve counterfactual reasoning. Under these circumstances, we require a model that can infer causes that are likely to be active for *out-of-sample* data (individuals with certain features, who may not have received any treatment yet), as opposed to *in-sample *data (individuals whose clinical data a particular model was trained on, who might have showed a different response to different treatment strategies) [29].**

**This perspective raises the issue of how representative the individuals who make up a particular training dataset are of the general population (from whom future patients will be drawn). Importantly, concerns have previously been raised as to the effects of various sources of selection bias on the representativeness of participants in RCTs compared to the population at large [30]. Specific forms of selection bias that have been identified in RCTs for psychological disorders include exclusion of individuals with comorbidities (which for many some conditions may be more common than ‘pure’ presentation), selection of less severe cases (e.g. in psychotic disorders, where ability to consent and treatment compliance may be of heightened concern), or, conversely, application of minimum severity thresholds (e.g. in mood disorders, to reduce the likelihood of spontaneous remission over the trial period) [31,32]. Further, methods of recruitment to RCTs (particularly the requirement to self-select into trials) may influence the distribution of various psychological traits in trial participants, prior to any further eligibility criteria being applied (e.g. [33]). Although there are methods designed to mitigate the effects of sample selection bias when transferring predictive models to a different test set (see [34]), it remains an open question as to whether these are sufficiently robust for successful out-of-sample treatment prediction at the individual level.**

**The success of causal inference modelling approaches to response prediction may therefore depend upon availability of different kinds of data to that derived from traditional RCTs – involving semi-continuous measurement of the relevant clinical outcome (both pre- and post- intervention), and gathered from more representative sources than some previous RCT datasets.** Given sufficient attention to patient confidentiality and other ethical concerns, it may be possible to obtain appropriate training data from health service clinical records; however, frequency and consistency of symptom reporting may pose analytical problems (e.g. 28). The use of personal devices such as smartphones or other wearable technology to regularly self-record symptom levels may be a potential source of this kind of data in the future, given sufficient insight and patient compliance (e.g. 35).

**A further important feature of predictive models derived from observational data is that they depend on explicit assumptions about the existence and causal consequences of any confounding variables present in the dataset. For example, the CGP approach requires both that there will be a consistency of outcomes between training observations and future outcomes, given a particular treatment, and that there are no important confounding variables missing from the dataset [27]. It will therefore be necessary to carefully consider how well such assumptions are met when considering applying these kinds of models to psychological and chronic pain symptom data.”**

# Peer review: Referee response for version 1

**DOI:** 10.5256/f1000research.14907.r29962

**Published:** 2018-02-02

**Authors:** William G. Hopkins

**Affiliations:** 1 Institute for Health and Sport, Victoria University, Melbourne, VIC, Australia

This article represents a valuable contribution to the developing literature on quantification of individual responses to treatments and identification of patients who respond positively to treatments. Perhaps there should be some attention to the issue of identifying negative responders, since it is more important to avoid harming individual patients than to miss out on benefitting them. By "harm" I don't mean side effects. I also point out in my review that prediction models could be developed from identified modifiers of the treatment effect in controlled trials, something that you should also mention. I also have a strong view about repeatability of individual responses to treatments, something that you will need to address by refuting my claim or accommodating it. Otherwise there are only minor points for you to consider. These were all made on a first and only read-through, so although you explain some points further on, it is important to avoid any confusion in the first place.

 "This may be particularly problematic in the case of psychiatric and chronic pain symptom data, where both within-subject variability and measurement error are likely to be high." I don't understand inclusion of "within-subject variability"? Are you referring to real changes of individual subjects pre to post the treatment that occur even in the absence of an active treatment? I normally think about that as another kind of measurement error. Perhaps you need to clarify by stating "both within-subject variability over the period of the treatment and short-term measurement error " Also, solitary "this" is a grammatical error known as an ambiguous antecedent. There are a few other instances in the manuscript that need to be fixed.

 "especially in arenas where the potential clinical benefit is so large" I don't understand the use of "so". Are you referring to the arenas of psych disorders and chronic pain? Are the "potential clinical benefits" in these arenas any larger than in any other arenas of pathology? And you haven't established (in the Abstract, anyway) that there is the potential for large benefit when individual responders have been characterised. Maybe something along the lines of "trustworthy identification of characteristics of positive responders to treatments could result in substantial clinical benefit in psychological disorders, chronic pain, and other pathologies."

 In the Introduction, you make it clear–at some length–that non-responders to one kind of treatment could be responders to another, and it may therefore be possible to improve the health of the majority of patients by targeting specific patients with specific treatments, where the pathology has a range of underlying causes and a range of treatments is available. Maybe you need to make that clearer in the Abstract.

 "variables that may potentially relate" is a "double doubtful." Remove either "may" or "potentially".

 "i.e. the reliability of distinguishing between treatment ‘responders’ and ‘non-responders’". I think validity would be a better word than reliability. Also no need for quote marks.

 e.g. and i.e. normally have commas after them and are used only in parentheses.

 "Crucially, we can only draw this inference by direct comparison…" Make it "Crucially, we can draw this inference only by direct comparison…" Check for any other instances of this misplaced modifier.

 You tend to use too many parenthetical asides. Remove parentheses from as many as you can.

 "Formally, to properly infer whether a particular participant responded or didn’t respond to a particular treatment, we would require knowledge of what would have happened if a key event (treatment administration) both did and did not occur (a form of counterfactual reasoning), which is not possible in the real world." I found this sentence confusing. What is counterfactual reasoning? If I understand this sentence correctly, I disagree with it. In crossovers it is possible to determine the outcome with an individual who received the active and the control treatment. You go on to state that yourself.

 "(e.g. time of day, stress level, etc.)" Either e.g. or etc., but not both!

 "Measurement error may be higher than [in] other areas of medicine, as the main tools used to assess clinical outcomes are patient or clinician-completed questionnaire measures, which are relatively low[-]precision tools." I think this statement is false, so you'd better support it with references. The square root of the alpha reliability (which provides an upper limit to the criterion validity correlation of multi-item instruments) and short-term retest reliability ICC could well be high enough to reasonably identify responders to short-term treatments. Even VASs have high short-term ICCs. The ICCs over periods for long-term treatments are likely to be a different story, as you point out.

 "If the variation in outcome due to these sources is greater than that due to any true individual differences in treatment response, it will be very hard to detect the latter under a conventional RCT framework." It depends what you mean by "detect".  To be on the safe side, perhaps you should state "The greater the variation in outcome due to these sources compared with that of true individual responses, the harder it will be to characterize the latter in a controlled trial." Note that I have removed superfluous words. Bottom line is that you can make up for the short- and log-term errors with a big-enough sample size, at least for characterizing the mean effect and its modifiers.

 "The fact the t1 score is used to calculate both quantities…" I fully understand regression to the mean, but I re-read this paragraph and still don't know what "both quantities" refers to.

 "The ability to properly identify differential response to a particular treatment in different individuals requires replication at the level at which the differential response is claimed (i.e., that particular treatment in that particular individual)." This rather obscurely worded claim has been made by others, but it is false. You can have a patient who responds individually to a treatment on one administration of the treatment. Whether that patient would respond similarly again following washout and reapplication of the treatment is irrelevant. What matters is that the patient has obtained benefit from the treatment when it was applied the first time. Period. Whether you can adequately quantify the extent of an individual patient's response to the (first) application of the treatment depends on short- and long-term errors of measurement and on the magnitude of the response in that patient, but regardless, you can certainly characterise modifiers of the treatment effect with realistic sample sizes: 4x the sample size required to characterize the mean effect (Hopkins, 2006). The identified modifiers could then be used to build courses-for-horses (treatments-for-patients) prediction models, something you haven't considered. Anyway, there is no need to confuse everyone by raising the spectre of repeatability of the treatment effect in individuals.

 "However, these may require additional measurements (e.g. multiple estimates of t1 value, in order to control for effects of measurement error)." No, repeating the t1 assessment reduces but does not eliminate the effect of regression to the mean. What you need for the adjustment is the reliability ICC over the time-frame of the treatment. In fact, the control group effectively provides that: when you predict the likelihood of an individual's response in a controlled trial using a mixed model in which the pre-test (t1) is included as a modifying covariate, you have controlled for (adjusted away the effect of) regression to the mean.

 I don't understand the paragraph headed Counterfactual probabilistic modelling. You will have to explain what is going on here without the jargon, for my benefit and for those who are even less statistically savvy.  I see the word "trajectory" in there, which suggests to me that you are talking about clinical trials with multiple repeated measurements during the course of the treatment. That's a luxury that may not be available in many settings, and in any case, it requires an appropriate model for the time course, which is bound to be non-linear. You still need a control group, if you want to eliminate the contribution of the placebo effect.

 Ah, I see you provide some explanation in the next paragraph. Please make the preceding paragraph clearer.

 "The CGP approach also rests on two key mathematical assumptions: that there will be a consistency of outcomes between training observations and future outcomes, given a particular treatment…"  No. See above.

 "and that there are no important confounding variables missing from the dataset." Be more explicit. If it's a properly balanced controlled trial (or randomized with a sufficiently large sample size), what's the problem?

 "One solution to this problem is to use data derived from repeated cross-over design clinical trials…" Well, no, because it introduces what I called above the spectre of repeatability.

 "It may be possible to alleviate these issues with careful model design…" Explain "careful". You will need to have identified the point(s) explaining "careful" previously, as this sentence is in the Conclusions.

 Hopkins, WG. Estimating sample size for magnitude-based inferences. Sportscience 10, 63-70 ( http://www.sportsci.org/2006/wghss.htm)

I have read this submission. I believe that I have an appropriate level of expertise to confirm that it is of an acceptable scientific standard, however I have significant reservations, as outlined above.

# Response

**Published:** 2018-02-27

**Authors:** Agnes Norbury

**Affiliations:** Department of Engineering, University of Cambridge, UK

Thank you for taking the time to review this manuscript. Please see below for responses to your main comments, which are addressed in the revised manuscript.

1. We have added reference to the possibility that appropriately formulated data-driven models could be used to predict probability of harm (symptom increase above a clinically significant threshold), as well as probability of successful treatment response (symptom decrease above some clinically significant threshold).

2. We have added reference to methods for the identification of treatment ‘modifiers’, whilst controlling for other artefacts, under a traditional statistical approach – plus a discussion of an analogous approach from the field of machine learning (please see below):

“It also may be important to think carefully about the nature of the predictors (features) included in supervised learning model training data – as those that reference initial clinical severity may be vulnerable to regression to the mean-related artefacts. **Under a traditional statistical framework, an effective way of dealing with these artefacts is to include baseline scores as covariates in models of symptom change data (i.e., conduct an ANCOVA). This approach can then be used to test if a given between-subjects variable is a significant modifier of treatment effect by adding it to the model (providing measurement error is sufficiently low, [24]). An interesting issue is that in machine learning, there is not really an equivalent concept to ‘covariates of no interest’ – rather, model features are usually selected purely on the basis of their predictive capacity. One recent paper that explicitly addresses this problem comes from Rao and colleagues, who propose a method for removing known confounds from predictive models based on functional imaging data. Rao et al. suggest that one solution is to first fit linear models to each image feature using the *confound* variables as predictors, then consider the residuals of this model to be ‘adjusted’ data − suitable to be used as input features for a confound-controlled predictive model [26]**.”

3. Regarding the issue of ‘repeatability’, we would argue that since this perspective deals with the ability to predict the responses of *future* patients (i.e., individuals with similar characteristics to past ‘responders’), then repeatability of these responses is not only relevant, but vital. Although we would not deny the real benefit to an individual patient of any significant improvement in clinical outcome, if this benefit is not ‘repeatable’ in that it is reliably casually related to treatment administration (e.g. score improvement is largely due to fluctuations in symptoms that would have occurred otherwise), then it should not be taken as evidence for use of that treatment in future (similar) individuals.

4. Some changes to grammar have also been made throughout the manuscript in order to increase clarity.

# Peer review: Referee response for version 1

**DOI:** 10.5256/f1000research.14907.r29856

**Published:** 2018-01-18

**Authors:** Greg Atkinson, Philip J. Williamson

**Affiliations:** 1 Health and Social Care Institute, Teesside University, Middlesbrough, UK; 2 Health and Social Care Institute, Teesside University, Middlesbrough, UK

In this manuscript, the authors discussed the concept of inter-individual differences in response to treatment interventions, particularly those focussed on psychological-related outcomes. The consideration of inter-individual responses is an important issue and the authors provide further insights previously not considered in detail within the domain of psychology. The topic is generally discussed accurately in the context of the current literature, statements are generally correct and supported by relevant citations. I have thoroughly read and considered the manuscript, which was interesting in content and constructed with a logical flow. I have only minor comments for the authors’ consideration. 

  

1. The article focuses on the prediction of response heterogeneity, especially prediction of responders/non-responders. Have you considered the roadmap that has been suggested ^1^ to actually confirm whether the general amount of true heterogeneity is clinically important or not BEFORE we might explore for predictors of that heterogeneity?
2. Page 3, Right hand Column, Lines 27 onwards– whilst discussing RCT trial design and highlighting that responders/non-responders cannot be identified through the analysis of intervention sample data alone, perhaps it might be appropriate to address any research making similar claims even in the total absence of any control sample data.
3. Page 4, Left hand column, Line 8 and conclusion. The arguments that you make on this point, particularly in the conclusion are, at present, unsupported by scientific literature and require justification. You allude to the fact that a lack of ‘true’ counterfactual information makes an RCT in effect a single-arm (no control study). It is agreed that one cannot say with 100% certainty whether the intervention group as a whole or any specific individual in the intervention group is a positive responder, as what would have happened to that person if they had been in the control group is of course unknown. This is the fundamental counterfactual basis of the RCT. Nevertheless, as the control group variability over the same time period as the intervention effectively provides our best guess of the counterfactual (what would have happened to individuals in the intervention group if they had been in the control arm), I feel that this applies to changes at both the group mean and the individual level, and that disregarding RCTs as ‘single-arm studies’ is unsupported. According to the previously-mentioned “roadmap” that has been presented, the analysis of the control group changes (specifically the comparison of change variance between treatment and control) can provide information as to what the general clinical importance is of “true” individual response heterogeneity. By “true”, one knows from this comparison whether the overall amount of heterogeneity in changes surpasses the overall amount of random within-subject heterogeneity of changes in the control group. If heterogeneity of change is similar between treatment and control, it could be argued that moving on to attempts to predict treatment response variability is a somewhat meaningless exercise.
4. Page 4, Left hand column, Lines 43 – 54. Whilst discussing regression to the mean and the mathematical coupling of pre- to post change scores, the use of covariates (especially baseline values of the study outcome) in the statistical model (ANCOVA) could be suggested as a potential solution to this – a notable absence in many studies’ data analyses.
5. Pages 4 – 5. You make a number of pertinent suggestions for potential solutions to the problem, and briefly allude to the methods recently suggested ^(1,3)^. We have suggested how this might be approached in RCTs and tied to an appropriate anchor usually a minimal clinically important difference or smallest worthwhile change ^(1,2)^. Addressing these issues may assist the reader in applying this methodology in their applied practice and/or research environments.

We have read this submission. We believe that we have an appropriate level of expertise to confirm that it is of an acceptable scientific standard.

## References

[1] AtkinsonGBatterhamAM: True and false interindividual differences in the physiological response to an intervention. *Exp Physiol*.2015;100(6) : 10.1113/EP085070 577-88 10.1113/EP085070 25823596

[2] WilliamsonPJAtkinsonGBatterhamAM: Inter-Individual Responses of Maximal Oxygen Uptake to Exercise Training: A Critical Review. *Sports Med*.2017;47(8) : 10.1007/s40279-017-0680-8 1501-1513 10.1007/s40279-017-0680-8 28097487

[3] HopkinsWG: Individual responses made easy. *J Appl Physiol (1985)*.2015;118(12) : 10.1152/japplphysiol.00098.2015 1444-6 10.1152/japplphysiol.00098.2015 25678695

# Response

**Published:** 2018-02-27

**Authors:** Agnes Norbury

**Affiliations:** Department of Engineering, University of Cambridge, UK

Thank you for taking the time to review this manuscript. Please see below for responses to your main comments, which are addressed in the revised manuscript.

1. We have added reference to the proposed ‘roadmap’ to determine whether clinically important response heterogeneity exists prior to quantifying it.

2. Regarding the reference to ‘single arm’ studies, we have made it more explicit that although the underlying RCTs (in order to determine treatment effectiveness as the group level) are not single arm, machine learning algorithms concerned with predicting individual differences in treatment response are usually only trained on the active treatment arm data (please see below):

“ **Although the underlying RCT datasets almost always consist of at least two arms (e.g. active treatment *vs* placebo), machine learning algorithms employed to predict psychiatric treatment response are usually trained on active treatment arm data alone – without reference to control arm symptom changes (e.g. [13,14]). Unless sufficient care is taken, these kinds of predictive models may therefore be the inferential equivalent of single arm trials, and the resultant categorisation of symptom change scores may be unduly influenced by sources of variance causally unrelated to true treatment response.**”

3. We have added reference to the use of ANCOVA under a traditional statistical framework as a way of guarding against regression to the mean and mathematical coupling artefacts – with discussion of equivalent techniques (or their absence) in the machine learning literature (please see below):

“It also may be important to think carefully about the nature of the predictors (features) included in supervised learning model training data – as those that reference initial clinical severity may be vulnerable to regression to the mean-related artefacts. **Under a traditional statistical framework, an effective way of dealing with these artefacts is to include baseline scores as covariates in models of symptom change data (i.e., conduct an ANCOVA). An interesting issue is that in machine learning, there is not really an equivalent concept to covariates of no interest – rather, model features are selected purely on the basis of their predictive capacity. One recent paper that explicitly addresses this issue comes from Rao and colleagues, who propose a method for removing known confounds from predictive models based on functional imaging data. Rao et al. suggest that a potential solution is to first fit linear models to each image feature using the *confound* variables as predictors, then consider the residuals of this model to be ‘adjusted’ data − suitable to be used as input features for a confound-controlled predictive model [26].**
